# In situ micro-spectroscopic investigation of lignin in poplar cell walls pretreated by maleic acid

**DOI:** 10.1186/s13068-015-0312-1

**Published:** 2015-08-27

**Authors:** Yining Zeng, Shuai Zhao, Hui Wei, Melvin P. Tucker, Michael E. Himmel, Nathan S. Mosier, Richard Meilan, Shi-You Ding

**Affiliations:** Biosciences Center, National Renewable Energy Laboratory, Golden, CO 80401 USA; National Bioenergy Center, National Renewable Energy Laboratory, Golden, CO 80401 USA; Department of Agricultural and Biological Engineering, Purdue University, West Lafayette, IN 47907 USA; Department of Forestry and Natural Resources, Purdue University, West Lafayette, IN 47907 USA; Department of Plant Biology, Michigan State University, East Lansing, MI 48824 USA

**Keywords:** Lignin autofluorescence, Fluorescence lifetime imaging microscopy, Stimulated Raman scattering imaging, Lignin–carbohydrate complexes droplets

## Abstract

**Background:**

In higher plant cells, lignin provides necessary physical support for plant growth and resistance to attack by microorganisms. For the same reason, lignin is considered to be a major impediment to the process of deconstructing biomass to simple sugars by hydrolytic enzymes. The in situ variation of lignin in plant cell walls is important for better understanding of the roles lignin play in biomass recalcitrance.

**Results:**

A micro-spectroscopic approach combining stimulated Raman scattering microscopy and fluorescence lifetime imaging microscopy was employed to probe the physiochemical structure of lignin in poplar tracheid cell walls. Two forms of lignins were identified: loosely packed lignin, which had a long (4 ns) fluorescence lifetime and existed primarily in the secondary wall layers; and dense lignin, which had a short (0.5–1 ns) fluorescence lifetime and was present in all wall layers, including the cell corners, compound middle lamellae, and secondary wall. At low maleic acid concentration (0.025 and 0.05 M) pretreatment conditions, some of the dense lignin was modified to become more loosely packed. High acid concentration removed both dense and loosely packed lignins. These modified lignins reformed to make lignin–carbohydrate complex droplets containing either dense or loosely packed lignin (mostly from secondary walls) and were commonly observed on the cell wall surface.

**Conclusions:**

We have identified dense and loosely packed lignins in plant cell walls. During maleic acid pretreatment, both dense lignin droplets and loosely packed lignin droplets were formed. Maleic acid pretreatment more effectively removes loosely packed lignin in secondary walls which increases enzyme accessibility for digestion.

## Background

Plant biomass constitutes a substantial renewable energy source. In order to produce drop-in fuels from biomass, combined chemical and biological conversion processes are used to produce liquid fuels adaptable to the current internal combustion engine design [1]. One of the biomass conversion routes under consideration is the depolymerization of polysaccharides in plant cell walls followed by the microbial conversion of biomass sugars to ethanol [2]. Alternatively, these sugars can also be used to produce various hydrocarbon products through biological conversion processes. For all the biomass–energy conversion schemes, polysaccharides, including cellulose and hemicellulose, are utilized to produce sugars; whereas, lignin, which accounts for the second-most abundant polymer in biomass (approximately 20–25 % of dry weight), plays a negative role affecting overall biomass conversion efficiency. Due to lack of understanding of the physiochemical structure of lignin in the plant cell walls, the mechanisms of lignin impediment during the conversion process remain poorly understood.

The primary units of lignin are *p-*coumaryl, coniferyl, and sinapyl alcohols [3, 4] that form a complex structure, cross-linked with other cell wall polymers. The differences in lignin content between layers of plant cell walls is thought to be the result of the unique lignin synthesis processes during cell growth. Lignin monomers are synthesized inside the cell membrane and then translocated to the cell wall [5, 6], initiating polymerization using oxidative polymerization processes which start at the end of the cell growth stage [5, 7]. Polymerized lignin starts at the cell corners and compound middle lamella [5–7], which includes the middle lamella and the primary cell wall, and gradually extends into the secondary cell wall. The lignified secondary cell wall contains three sub-layers, denoted as S1, S2 and S3 (from the outside in); S1 and S3 are thin and S2 constitutes the majority of the secondary wall [8, 9]. Because lignin is associated with carbohydrate and distributed unevenly among different cell wall layers, the ideal technique to study lignin distribution on cell wall layers should offer chemical specificity, high spatial resolution, no need for exogenous labels, and non-invasiveness.

In the past, Raman micro-spectroscopy as a label-free microscopic technique has been widely used because it offers high spatial resolution and high chemical specificity [10–12]. However, the Raman scattering signal is intrinsically weak and often requires a long image acquisition time for a confocal Raman microscope to generate an image with clear pixel resolution. In the recently developed stimulated Raman scattering (SRS) microscopy, the Raman signal is linearly dependent upon the concentration (as in confocal Raman), and is enhanced by orders of magnitude through the stimulated excitation of molecular vibrational transitions [13, 14]. Previously, we reported applying SRS to the imaging of lignin in plant cell walls, with spatial resolution and fast image acquisition based on lignin’s unique strong aromatic Raman vibration band at 1600 cm^−1^ [15–18]. This approach requires no further treatment to lessen cell wall autofluorescence background. We have shown that in a typical poplar cell wall transverse section, the cell corner has the highest lignin concentration, followed by the compound middle lamella [18]. The secondary cell wall (in most of the cases, the sclerenchyma-type secondary wall) is also highly lignified, but most of the lignin is in the S2 layer. The S3 layer has moderately high lignin concentration, especially in the warty layers. However, due to its relative thinness, the total amount of lignin in S3 is lower than in S2 [18].

Besides Raman activities, lignin generates significant autofluorescence emission when excited by UV light. Fluorescence lifetime is the average time a fluorophore remains in an excited state upon excitation, which is dependent on several factors which include: the rate of spontaneous emission of fluorescence, the rate of internal conversion, the rate of intersystem crossing to triplet state, and the rate of resonance energy transfer. Fluorescence lifetime is therefore determined not only by the chemical structure, but also by the local environment of the fluorophores. This distinction allows fluorescence lifetime measurement to probe the molecular environment of fluorophores or labeled molecules in biological systems [19–22]. Fluorescence lifetime imaging microscopy (FLIM) generates an image from a fluorescent sample where each pixel measures the average lifetime from the corresponding focus spot. FLIM of lignin autofluorescence provides a spatial distribution of the fluorescence lifetime decay rate of lignin in plant materials [23–25]. Combining SRS and FLIM together will provide a correlative imaging approach to probe both chemical composition and lignin structure in the same location of the cell walls in situ. Because cell wall tissues are usually fragile after pretreatment, our label-free imaging approach requires no additional sample preparation, and therefore provides for the direct investigation of pretreated samples.

The fully lignified walls from tracheary cells represent the majority of the mass in woody materials. Lignin is chemically bonded with hemicellulose using ester, ether, and glycosidic bonds to form the lignin–carbohydrate complexes (LCC) barrier that acts as a protective layer over cellulose in plant cell walls, preventing them from being attacked by cellulase enzymes [3, 26]. Non-covalent interactions may also link lignin and hemicellulose, but there are few interactions reported between lignin and cellulose [26]. For the enzyme-based biomass saccharification process, pretreatment is required to break the LCC barriers and expose cellulose surfaces for effective enzyme access [2]. It has been well documented in the literature that the total amount of lignin in raw biomass is usually negatively correlated with enzymatic digestibility [27, 28]. However, in practice, pretreatment processes targeted at nearly complete lignin removal are usually too expensive. Previously, we reported that in addition to the amount of lignin, the redistribution of lignin on biomass after pretreatment is also an important factor that affects enzymatic digestibility [28]. It has been suggested that for enhancing enzymatic digestion of cellulose, the ideal pretreatment should maximize lignin removal and retain the cellulose microfibril structure [29]. Therefore, the highly efficient and cost-effective pretreatments should modify the right amount of lignin at the best location in the wall, but still allow maximum enzyme accessibility and digestion [28]. Over the past several decades, many pretreatment approaches have been developed. Among these, dilute acid pretreatment has been extensively studied, especially in grassy feedstocks. Dilute acid pretreatment hydrolyzes hemicelluloses and thus delocalizes LCCs and opens the cellulose surface for subsequent enzymatic hydrolysis. However, under acidic conditions and elevated temperatures, dilute acid pretreatment, such as the widely used sulfuric acid, results in degradation of a significant fraction of xylose to furfural and some glucose to hydroxymethyl furfural, levulinic acid, and formic acid [30, 31], which reduces sugar yields and generates inhibitory compounds to microbes during fermentation [32–35]. Inspired by biomimetic catalyst design, dicarboxylic acids, such as maleic acid, have been considered because of their chemical structural similarity to that of the catalytic sites of cellulases, which employ two amino acid residues, acting as a proton donor and nucleophile pair, to hydrolyze glycosidic bonds via acid catalysis mechanism [36]. Previously, maleic acid has been demonstrated to achieve higher glucose and xylose yield than sulfuric acid pretreatment, due to lower sugar degradation [37] during pretreatment. This is because maleic acid is more active in breaking down xylan into xylose and less active in degrading xylose to furfural [38, 39]. An economic analysis of the pretreatment alone shows that the optimized diluted maleic acid pretreatment costs 65 € per metric ton dry wheat straw. One key factor is the high cost of the maleic acid itself [40]. Its high cost may be partly compensated by higher return on xylose yield, reduced sugar loss to degradation and less capital cost due to its less corrosive nature [36–39, 41]. A combined biomimetic and inorganic acids pretreatment could also lower the cost of acids while still maintaining high yield [42]. Despite extensive kinetic studies on maleic acid-catalyzed carbohydrate hydrolysis, microscopic investigation on how maleic acid pretreatment impacts lignin in the cell walls has not been reported. A better understanding of the mechanisms of lignin modification during pretreatment provides valuable insights into new pretreatment strategies.

Poplar has received significant attention recently as a fast-growing energy crop widely available on fallow lands to provide an ample supply of biomass [4, 43, 44]. Poplar has been regarded as a model hardwood species for breeding modified genotypes for bioenergy applications [45]. The tracheary cell walls that are predominant in poplar possess relatively uniform chemical composition among the cells [6, 18]. This provides a reliable basis for comparative microscopic studies to represent overall biomass pretreatment. In addition, maleic acid has been previously demonstrated to be an effective pretreatment compared to dilute sulfuric acid. Here, we apply our unique micro-spectroscopic system combining FLIM and SRS microscopy to investigate the in situ lignin concentration and structural change in untreated and maleic acid-treated poplar cell walls.

## Results and discussion

### Dense and loosely packed lignins in untreated poplar cell walls

**Fig. 1:**
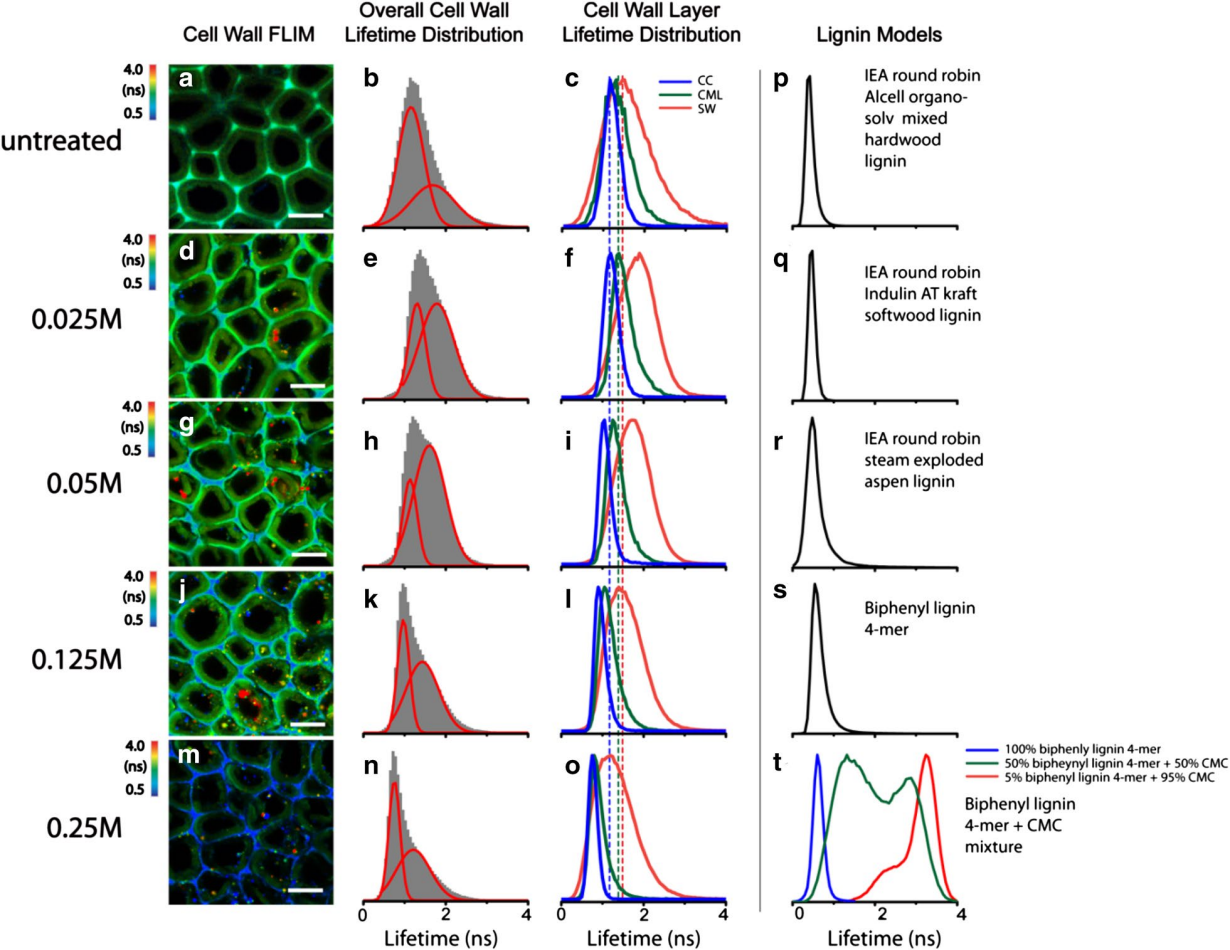
FLIM analysis of lignin fluorescence lifetime in poplar cell walls. Cell wall FLIM are representative FLIM images of cell wall-based on lignin autofluorescence with 405 nm laser excitation, including untreated (**a**) and treated with different concentrations of maleic acid (**d**, **g**, **j**, **m**) indicated on the *left*. *Scale bar* 10 μm. Overall cell wall lifetime distribution (**b**, **e**, **h**, **k**, **n**) shows the overall lignin fluorescence lifetime distributions from all the cell wall layers. The *two red curves* are the two fitted Gaussian peaks by fitting the overall histogram. They represent the fluorescence lifetime distributions of dense and loose lignin in cell walls. Cell wall layer lifetime distribution (**c**, **f**, **i**, **l**, **o**) shows the individual cell wall layer (*CC* cell corner, *CML* compound middle lamella, *SW* secondary wall) lignin fluorescence lifetime distributions. Lignin models show the fluorescence lifetime distributions of lignin model compounds. Each individual four model compounds (**p**–**s**) and mixtures of biphenyl lignin 4-mer and carboxymethyl cellulose (CMC) at different ratios (**t**)

The preponderant cells in the poplar xylem are tracheary elements that have secondarily thickened walls that on average are about 3 µm thick. Figure 1a shows a typical FLIM image of untreated latewood tracheid, based on the autofluorescence of lignin generated by 405 nm excitation. The overall fluorescence lifetime distribution of all cell wall locations (including the secondary cell wall, cell corners, and compound middle lamella) is shown in Fig. 1b. This broad fluorescence lifetime distribution is an apparent non-Gaussian distribution that contains short lifetime components centered at 1 ns and long lifetime components centered at 2 ns (Fig. 1b, red curves). Clearly, the autofluorescence lifetime of lignin across the cell wall is not evenly distributed among wall layers. Lignin in the secondary cell wall has a slightly longer lignin fluorescence lifetime and a broader distribution than those in the cell corner and compound middle lamella. Lifetime distributions of cell corner, compound middle lamella, and secondary cell walls from the same untreated sample (Fig. 1c, curves CC and CML) mostly contain short lifetime components, and the secondary cell wall contains both short and long lifetime components (Fig. 1c, curve SW).

In order to further understand the correlation between apparent lignin autofluorescence lifetime and its physiochemical structure in the cell walls, lignin model compounds obtained from various sources have been investigated as controls using FLIM. Four lignin compounds were used in this study including three IEA lignins extracted from different plant biomass as described previously that contain almost pure lignin with little carbohydrate content [46, 47] and the 4-mer biphenyl lignin that was synthesized. Interestingly, despite the chemical variations of these lignin compounds, they consistently demonstrate narrow fluorescence lifetime distributions centered at 0.5–1 ns (Fig. 1p–s), indicating that fluorescence lifetime is a reflection of lignin concentration that seems to be independent of the sources from which the lignin is obtained. To verify that hypothesis, artificial lignin–carbohydrate composites were prepared by co-precipitation of a biphenyl lignin 4-mer (one of the lignin model compounds) and carboxymethyl cellulose (as the carbohydrate) from solution. The residual moisture was removed by vacuum and incubated overnight at 90 °C. The lignin–carbohydrate complex thin film was then imaged under FLIM to obtain the fluorescence lifetime distribution across the film. We found that increasing carbohydrate content in the complex leads to the increase of lignin fluorescence lifetime. The composite containing 50 % lignin and 50 % carbohydrate showed very broad fluorescence lifetime distribution from 1 to 3 ns (Fig. 1t, green curve). The composite with very low lignin content (5 % lignin and 95 % carbohydrate) showed a sharp distribution fluorescence lifetime centered around 3.5 ns (Fig. 1t, red). These observations suggest that the lower the lignin concentration, the longer its fluorescence lifetime. A control sample containing 100 % lignin model compound prepared using the same method was identical in fluorescence lifetime (Fig. 1t blue and Fig. 1s), indicating that the sample preparation process does not alter the fluorescence lifetime of lignin.

**Scheme 1:**
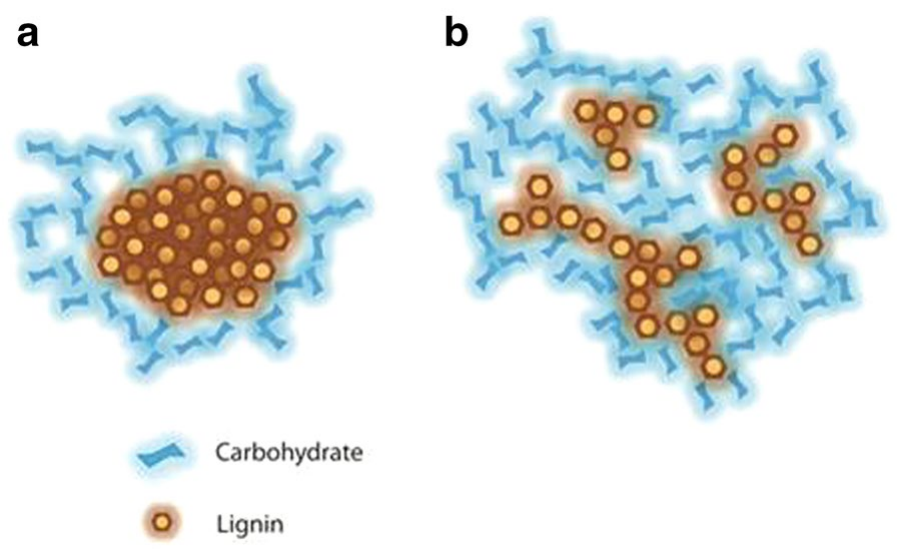
Hypothetical depictions of structures of **a** the dense lignin consisting primarily of aggregates of lignin with few carbohydrates on the surface, and **b** the loosely packed lignin consisting primarily of carbohydrate cross-linked with a small amount of lignin

In untreated cell walls, the lignin components that have short and long fluorescence lifetimes may correspond to the dense (or concentrated, Scheme 1a) and the loosely packed (or dilute, Scheme 1b) lignins, respectively. Hereafter, we refer to the lignin with a short fluorescence lifetime as “dense lignin”, and the long fluorescence lifetime lignin as “loosely packed lignin”. We further suggest that the dense and loosely packed lignins in untreated poplar cell walls are the result of the two lignification stages occurring during plant cell development, namely the I- and S-lignification processes [48]. As reported previously, the I-lignification occurs during an early stage of secondary cell wall thickening, mostly at the cell corners, which has a relatively high concentration of lignin monomers and peroxidases in an open space devoid of cellulose microfibrils. In this space, lignin is formed and adhered firmly between neighboring cells. I-lignification produces mostly dense lignin with the short fluorescence lifetime observed at the cell corner. In compound middle lamella, which is composed of middle lamellae containing no cellulose and the thin primary cell wall containing mostly cellulose macrofibril networks with large pores, lignins appear to have slightly longer lifetimes than in the cell corner. In general, the cell corner and compound middle lamella contain dense lignin with a relatively short fluorescence lifetimes. During S-lignification, which occurs after the development of secondary cell wall framework, lignin precursors permeate into the cellulose microfibrils framework mainly in the S2 layer of the secondary cell wall. Compared to I-lignification, S-lignification produces relatively smaller amounts of lignin associated with large amounts of cell wall hemicellulose. This type of lignin is the less concentrated “loosely packed” lignin showing longer fluorescence lifetime.

### How does maleic acid affect cell wall lignin and its micro-environment?

It has been reported that maleic acid as a catalyst for the pretreatment of various biomass achieves the most efficient xylan to xylose conversion under the optimum maleic acid concentration ~0.2 M with temperature at ~160 °C [38, 39, 49]. Lower maleic acid concentration will lead to lower xylose yield [38] and a higher pretreatment temperature (>180 °C) will cause significant xylose dehydration to furfural [49]. In this study, we choose the optimum pretreatment temperature (160 °C) and explore the maleic acid pretreatment effect on cell wall lignin distribution with a variety of maleic acid concentrations.

Figure 1 (Cell Wall FLIM) presents the representative FLIM images of poplar tracheid cell walls pretreated by maleic acid. Delamination of cell wall layers occurs at low acid concentration (0.025 M) [39], and separation of secondary walls and compound middle lamellae occurs in some cells (Fig. 1e). Increasing acid concentration leads to more cell wall separation (Fig. 1g, j, m). The spatial separation of the dense and loosely packed lignin distributions in cell walls, presenting as short and long fluorescence lifetimes, respectively, in the FLIM images is more obvious after maleic acid treatment. Elevating acid concentration from 0.025 to 0.25 M, led to an increase in the population of dense lignin that extends from the cell corners to compound middle lamella (Fig. 1g, j, m). The loosely packed lignin becomes spatially confined to secondary cell wall after treatment using high acid concentration (0.25 M).

We further analyzed the lifetime distribution from the whole cell wall (Fig. 1b, e, h, k, n) and in each wall layer (e.g., cell corner, compound middle lamella, and secondary wall (Fig. 1c, f, i, l, o). Compared to the untreated cell wall, pretreatment at low maleic acid concentrations (0.025 and 0.05 M) appeared to increase the relative amount of loosely packed lignin. At high maleic acid concentrations (0.125 and 0.25 M), the total amounts of lignin in the cell walls were significantly reduced, and more dense lignin remained in the cell wall (Fig. 1l, o), indicating that most of the loosely packed lignin was extracted [50–52] when hemicellulose was mostly hydrolyzed at high acid conditions [37–39].

In the secondary wall, treatments with low maleic acid concentrations (0.025–0.05 M) led to increases of lignin fluorescence lifetime (Fig. 1f, i, red curves), suggesting that lignin is modified when hemicelluloses [50, 53, 54] are partially hydrolyzed at low acidic conditions, causing modification of some of the dense lignin to become looser. It is well known that some of lignin can be dissolved under acidic conditions. Ether or ester bonds linking lignin and polysaccharides can be cleaved forming hydroxyl, carbonyl, or carboxyl groups, producing small molecular weight lignin fragments [50–54]. Depolymerization of hemicelluloses in the cell wall by acid may also cause some dense lignin unfolding and further reaction with polysaccharides to form loosely packed lignin. A further increase of acid concentration leads to decrease of loosely packed lignin in the cell wall (Fig. 1k, n), indicating that loosely packed lignin–hemicellulose complexes can be further fragmented and eventually extracted from the cell wall.

Together, those results support the hypothesis that at low maleic acid concentration, some dense lignin may be modified to produce a greater amount of loosely packed lignin. At higher maleic acid concentrations, the lignin that remains in the cell wall is mostly dense lignin, which could be due to the recondensation of lignin fragments during pretreatment or the hydrolysis of hemicelluloses.

Whereas lignin in secondary cell walls seems to be impacted differently by maleic acid treatment depending on acid concentration, in the cell corner and compound middle lamella, lignin fluorescence lifetime consistently decreases (Fig. 1f, i, l, o, blue and green curves) when acid concentration increases. This result is similar to that of the lignin model compounds (Fig. 1p–s) after high acid treatment and indicates that lignin at the cell corner and compound middle lamella may undergo condensation via carbonium ions and nucleophiles to form denser lignin [50, 53] when pectin is hydrolyzed and extracted [55] by maleic acid.

### Formation of LCC droplets by maleic acid pretreatment

**Fig. 2:**
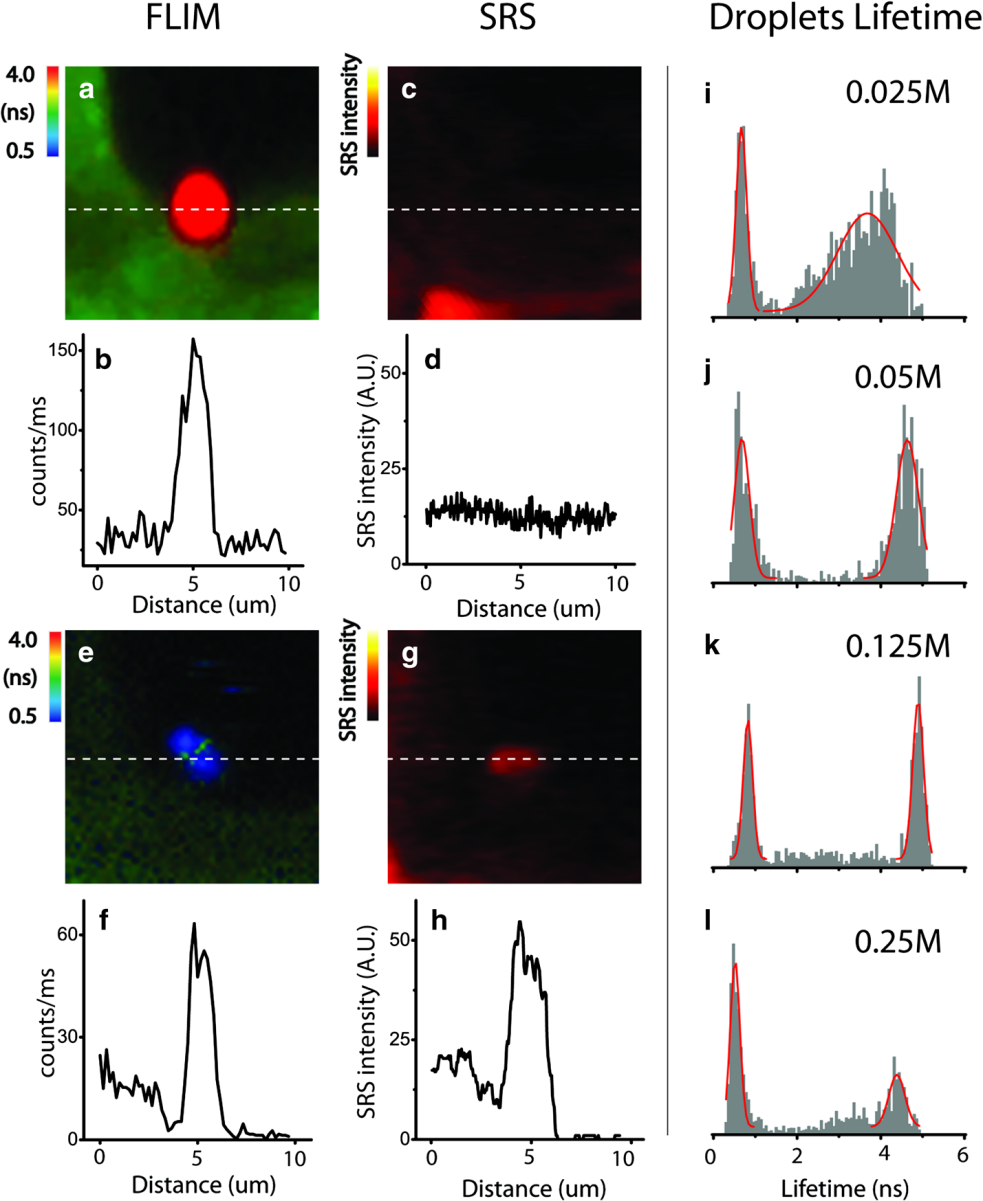
Comparison of FLIM and SRS micrographs in the same lignin droplets (**a**–**h**) and the overall fluorescence lifetime of lignin droplets after maleic acid treatment (**i**–**l**); droplets lifetime shows the overall lifetime distribution based on analysis of ~50 droplets formed under various maleic acid pretreatment concentrations. Consistently there are bimodal distributions of fluorescence lifetime that correspond to the dense and loosely packed lignin droplets. At low acid concentration (0.025 M) treatment the droplets are more diversified than at high acid concentration. Representative droplets containing loose lignin (**a**–**d**) and dense lignin (**e**–**h**) are analyzed by FLIM and SRS imaging at the same location. Line-scans are also presented under each image to show the relative intensity. Image size is 10 μm for all images

Globular droplets have long been observed following various pretreatments [56–60]; however, their content and formation mechanism are still under investigation. The FLIM images show that despite their broad size distribution (hundreds of nanometers to several microns in diameter), the binary fluorescence lifetime distribution collected from an ensemble of droplets shows that they are primarily two types of droplets: the droplets containing dense lignin showing a short fluorescence lifetime of 0.5–1.0 ns and the droplets containing loosely packed lignin showing a long fluorescence lifetime of 4.0 ns and longer (Fig. 2i–l). Compared to the fluorescence lifetime distribution of the lignin model compounds studies (Fig. 1p–t), the dense lignin droplets are composed of mostly lignin, whereas the loosely packed lignin droplets have rather low lignin concentration.

The SRS images were collected at lignin’s resonance frequency at 1600 cm^−1^, in which the intensity of the SRS signal is linearly correlated with lignin concentration. Generally, lignin droplets that have a long fluorescence lifetime show low lignin concentration. Figure 2a, c show one representative loosely packed lignin droplet with ~4 ns fluorescence lifetime. The same droplet appears to have a very low lignin concentration that is undetectable by SRS (Fig. 2c). Dense lignin droplets that have a short fluorescence lifetime (e.g., 0.7 ns in Fig. 2e, f) show high lignin concentration in the SRS image (Fig. 2g, h). Interestingly, the LCCs containing dense lignin appear to be similar in lifetime distribution to the lignin in the cell corners and compound middle lamella. Although during the pretreatment, dense lignin can be obtained by the recondensation of lignin fragments, it is also possible that the dense lignin LCCs originate from these cell wall layers. It could also be further speculated that the loosely packed lignin droplets are mostly from the secondary cell wall after acid treatment. However, some loosely packed lignin droplets appear to have a longer fluorescence lifetime (~4 ns) than those in the secondary wall, indicating that lignin is modified after it is extracted from the cell walls. After treatment at high maleic acid concentration, most hemicelluloses are hydrolyzed, which is evidenced by the significant reduction in the relative population of loosely packed lignin droplets and increases in the dense lignin (Fig. 2l vs. i–k).

### Removal of lignin in secondary cell walls is important for efficient enzymatic digestion

**Fig. 3:**
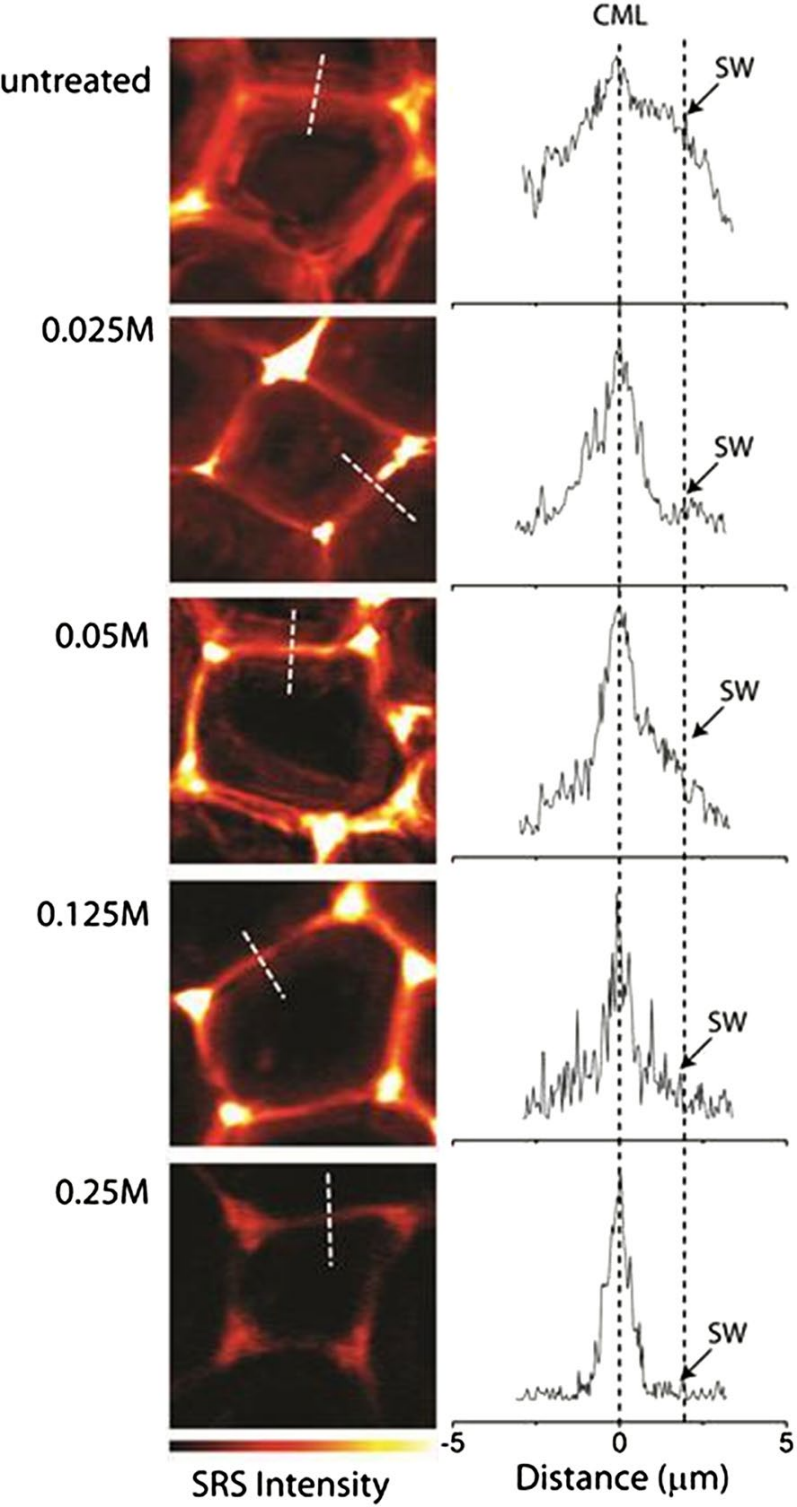
SRS images of lignin resonance from a typical polar cell wall before and after maleic acid pretreatment with different acid concentrations indicated on the *left*. The line-scan profiles across the cell wall (*dashed lines* in corresponding images in the left images) show that the lignin content at secondary cell wall (SW) is more reduced than compound middle lamella (CML)

It is clear that maleic acid pretreatment does not completely remove lignin from the cell walls. Even at the highest maleic acid concentration (0.25 M) employed in this study, which has been shown to produce good enzymatic digestibility [38, 39, 49], there is still a considerable amount of lignin left in the cell wall (Fig. 1m–o). This result is consistent with reported observations that total lignin removal is not necessary in order for efficient enzymatic hydrolysis of the cell wall. With SRS microscopy, we compared the lignin concentrations in different cell wall layers, e.g., the cell corner, compound middle lamella, and secondary wall, both untreated and treated at various maleic acid concentrations. The overall lignin distribution and line-scan across the cell wall layers are shown in Fig. 3. In the untreated sample, the secondary cell wall has substantial amounts of lignin; the increase of maleic acid concentration in treatment appears to lead to more lignin reduction in the secondary wall than in the cell corner and compound middle lamella. Eventually, most of lignin in the secondary cell wall is removed at 0.25 M maleic acid concentration, but there is still a significant amount of lignin left in the cell corners and compound middle lamella. The cell corner/compound middle lamella layers may be important for mass transportation during pretreatment in solution, although enzymes also penetrate from the cell lumen side of the wall during enzymatic hydrolysis [28]. Therefore, removal of lignin from the secondary wall is critical for the overall digestibility of the cell walls by enzymes.

## Conclusion

Based on in situ micro-spectroscopic analysis of lignins in the cell wall layers of poplar tracheary cells, the cell corners and compound middle lamella contain primarily dense lignin, and the secondary cell wall contains both dense and loosely packed lignins which are associated with hemicellulose. During maleic acid pretreatment, lignins are extracted from these cell wall layers and modified depending on acid concentration. The dense lignin located in the cell corners and compound middle lamella migrates from these zones and is released into solution, forming dense lignin droplets. The loosely packed lignin located in the secondary cell wall is released with hemicellulose, forming loosely packed lignin droplets containing relatively low lignin concentration. Maleic acid pretreatment appears to effectively remove loosely packed lignin from the secondary wall, thus achieving high digestibility by enzymes even though a significant amount of lignin remains in the cell corners and compound middle lamella.

## Methods

### Poplar samples

The wild-type *Populus tremula* × *P. alba* (cultivar INRA 717-1B4) was a regrowth of a 6-year-old tree that had been coppiced. The regrowth was cropped when it was about 1 year old, at 12 cm above the ground. This block was sectioned from the bottom of the main trunk, kept in −80 °C freezer for storage, and thoroughly rinsed with deionized (DI) water to remove soil and dust before use. A rotary microtome (Leica, RM2235) was used to transversely section the sample into 50-μm slices for pretreatment experiments.

### Lignin model compounds

International Energy Agency (IEA) Alcell organosolv-mixed hardwood lignin, IEA Indulin AT kraft softwood lignin, and IEA steam-exploded aspen lignin were the standard lignin model compounds carefully prepared for the IEA round robin tests for selected analytical methods to support the characterization of feedstocks. Researchers involved in this testing were from Canada, Finland, the Netherlands, New Zealand, Norway, Sweden, and the United States, and the results of the round robins were presented at the IEA Pre-symposium on Modern Methods of Analysis of Wood in 1991 [46]. Finally, the biphenyl lignin 4-mer lignin model compound was synthesized and characterized by high-performance size-exclusion chromatography from previous work [47].

### Maleic acid pretreatment of poplar samples

Maleic acid (ReagentPlus^®^, Sigma-Aldrich M0375) was purchased from Sigma-Aldrich. Solutions of various concentrations were prepared in deionized water.

The 50-μm poplar slices (approx. 0.025 g wet) were loaded into a 2-mL glass reaction vial containing 1 mL maleic acid solution (0.025, 0.05, 0.125 or 0.25 M) and sealed with a silicon cap. An aluminum plate was backed on top of the silicon cap to reinforce the sealing. The steam pretreatment was done on a two-gallon Parr reactor (Parr Instrument Co., Moline, IL, USA). The reaction vials were heated up to 160 °C and 77 psi in 60 s. Temperature and pressure were held steady for 10 min and then cooled down in 90 s to room temperature and ambient pressure. The pretreated poplar slices were rinsed by deionized water five times and placed between two #1 glass coverslips for microscopic imaging.

### Preparation of artificial composites containing lignin and carbohydrate

Carboxymethyl cellulose (Sigma C5678) was dissolved in 50 %:50 % ethanol:water to produce ~0.5 % solution. Biphenyl lignin 4-mer was then added to the above carboxymethyl cellulose solution to achieve biphenyl lignin 4-mer concentration 0.5 % (lignin:carbohydrate = 50 %:50 %) or 0.026 % (lignin:carbohydrate = 5 %:95 %). The solution was dropped on coverslip and vacuum-dried overnight to form a thin lignin–carbohydrate film. The thin film was then warmed up to 90 °C to remove residual solvent. As a control, the biphenyl lignin 4-mer thin film was prepared through the same procedure with 0.5 % biphenyl lignin 4-mer in 50 %:50 % ethanol:water.

### FLIM and SRS microscopy

FLIM and SRS microscope share the same microscope base—an Olympus IX71 inverted microscope with a high numerical aperture objective (UPlSApo 60X 1.20 NA W, Olympus). The FLIM system is PicoQuant MT200, equipped with 405 nm laser excitation at 40 MHz (PicoQuant LDH-P-C-405B). The epi-fluorescence signal was collected after a 430-nm long-pass filter (Chroma HQ430LP). The FLIM image was captured with 512 × 512 pixel resolution at 0.8 ms per pixel dwell time (~7 min per image). Laser intensity was kept at around 0.1 µW level to minimize photobleaching while still obtaining an adequate signal-to-noise ratio. FLIM images and lifetime analysis were performed in PicoQuant SynPhoTime (V5.3.2.2).

SRS microscopy was performed on the same equipment described previously [28]: a high-power Nd:YO_4_ oscillator (picoTRAIN, HighQ Laser, Austria) that produced 7 ps pulse trains at 1064 nm (15 W max) and 532 nm (9 W max). 2 W of the 1064-nm light was used as the Stokes beam. The 532-nm beam was directed to pump an optic parametric oscillator (Levante Emerald, APE GmbH, Germany) to produce a 6-ps tunable wavelength pulse train as the pump beam, and it was tuned to 909.2 nm for a lignin resonance frequency of 1600 cm^−1^. The Stokes beam was intensity-modulated by an acoustic optic modulator (3080-122, Crystal Technology) at 10 MHz with 80 % modulation depth and then combined with pump beams having a long-pass beam combiner (1064dcrb, Chroma). The two beams were routed to a custom-modified mirror-scanning microscope system (BX62WI/FV300, Olympus) attached with the Olympus inverted microscope as FLIM. Typical laser power at the sample plane was 80 mW for each beam, which allowed for continuous imaging without causing significant photo damage. The light transmitted through the sample was collected by a high numeric aperture condenser (1.45 NA O, Nikon) and filtered by an optical filter (CARS980/220, Chroma) to block the Stokes beam completely. This was done so that only amplitude modulation on the pump beams at 10 MHz (due to the SRS process) can be detected. Pump beam intensity was detected by a large area silicon PIN photodiode (FDS1010, Thorlabs) back-biased at 70 V. A lock-in amplifier (SR844, Stanford Research Systems) with full-scale sensitivity set at 100 µV was used to detect the intensity change in pump beam.

## Abbreviations

SRS: stimulated Raman scattering
FLIM: fluorescence lifetime imaging microscopy
LCC: lignin–carbohydrate complexes
CC: cell corner
SW: secondary wall
CML: compound middle lamella
CMC: carboxymethyl cellulose
